# Alterations in Muscle Architecture: A Review of the Relevance to Individuals After Limb Salvage Surgery for Bone Sarcoma

**DOI:** 10.3389/fped.2020.00292

**Published:** 2020-06-16

**Authors:** Christa M. Nelson, Victoria Marchese, Kelly Rock, Robert M. Henshaw, Odessa Addison

**Affiliations:** ^1^Department of Physical Therapy and Rehabilitation Science, University of Maryland School of Medicine, Baltimore, MD, United States; ^2^Department of Orthopedic Oncology, MedStar Georgetown Orthopedic Institute, Washington, DC, United States; ^3^Department of Orthopedic Oncology, Children's National Medical Center, Washington, DC, United States; ^4^Baltimore VA GRECC, Baltimore, MD, United States

**Keywords:** bone sarcoma, limb salvage surgery, muscle architecture, lower extremity, muscle adaptation, ultrasound, physical therapy

## Abstract

Osteosarcoma and Ewing's sarcoma are the most common primary bone malignancies affecting children and adolescents. Optimal treatment requires a combination of chemotherapy and/or radiation along with surgical removal when feasible. Advances in multiple aspects of surgical management have allowed limb salvage surgery (LSS) to supplant amputation as the most common procedure for these tumors. However, individuals may experience significant impairment after LSS, including deficits in range of motion and strength that limit function and impact participation in work, school, and the community, ultimately affecting quality of life. Muscle force and speed of contraction are important contributors to normal function during activities such as gait, stairs, and other functional tasks. Muscle architecture is the primary contributor to muscle function and adapts to various stimuli, including periods of immobilization-protected weightbearing after surgery. The impacts of LSS on muscle architecture and how adaptations may impact deficits within the rehabilitation period and into long-term survivorship is not well-studied. The purpose of this paper is to [1] provide relevant background on bone sarcomas and LSS, [2] highlight the importance of muscle architecture, its measurement, and alterations as seen in other relevant populations and [3] discuss the clinical relevance of muscle architectural changes and the impact on muscle dysfunction in this population. Understanding the changes that occur in muscle architecture and its impact on long-term impairments in bone sarcoma survivors is important in developing new rehabilitation treatments that optimize functional outcomes.

## Introduction

Bone sarcomas, particularly osteosarcoma and Ewing's sarcoma, are primary malignant musculoskeletal tumors affecting ~650 children and adolescents yearly in the United States with a 5-years survival rate of 65–75% for localized disease after treatment (1, 2). These tumors often occur near major joints and require extensive bone and soft tissue removal, restoration of a mechanically stable skeleton, and muscular or tendon reconstruction in order to salvage the limb (3). Although limb salvage surgeries (LSS) offer many benefits, extensive reconstruction and interruption of musculoskeletal structures likely contribute to lifelong functional impairments.

Survivors of childhood bone sarcomas demonstrate deficits of range of motion (ROM), strength, and increased pain (4–14). These impairments contribute to restrictions in gait, limb function, and physical activity ultimately affecting quality of life (QoL) (10, 15–20). Alterations in muscle function resulting from chemotherapy, surgery, and/or radiation likely impact muscle force production needed during activities of daily living.

Muscle architecture, defined as the size and orientation of muscle fibers in relation to the tendon of insertion, is known to be a primary determinant of muscle function (21). Fiber length contributes significantly to maximal contraction velocity, affecting the ability to generate muscle force quickly during functional activities (22). Muscle size, commonly measured via cross-sectional area or volume, also has significant contributions and correlates with muscle force (21, 23). Alterations in muscle architectural parameters have been observed in stroke, sarcopenia, cancer, cerebral palsy, and orthopedic conditions such as knee osteoarthritis and anterior cruciate ligament reconstruction (ACLR) (24–43). However, while much of this research has been in adult populations, studies of muscle architectural changes in postoperative pediatric and adolescent populations have been lacking. Architectural adaptations may significantly impact muscle force production and activities dependent on substantial force generation such as climbing stairs. Despite a clear link between muscle architecture and muscle function, little is known about the impact of LSS on muscle architecture. Given the unique medical and surgical management that these individuals undergo, multiple factors likely influence alterations in muscle architecture, contributing to functional and activity-related restrictions commonly observed.

The purpose of this review is to inform health care professionals on potential muscle architectural changes in individuals with LSS for bone sarcomas in order to optimize current rehabilitation strategies and highlight areas in need of further research. This review will focus on three areas: [1] current medical and surgical management of sarcomas and its impact on muscle, [2] measurement of muscle architecture and its impact on function, and [3] recommendations for future research in order to improve outcomes and optimize muscle and functional performance in long-term survivorship. Literature targeted for this review included peer-reviewed cross-sectional, epidemiological, longitudinal, and clinical studies in the pediatric, adolescent, and young adult population. Relevant studies in adult populations will be highlighted to inform readers on related research and its relevance to the population of interest.

## Lower Extremity Sarcomas and Surgical Management

### Diagnosis and Surgical Management of Lower Extremity Sarcoma

Individuals with bone sarcomas are frequently diagnosed in the 2nd decade of life, at a time of continuing development and growth (3, 44, 45). A majority of these bone sarcomas occur in the appendicular skeleton, especially in the femur or tibia adjacent to the knee (45, 46). Management of bone sarcomas includes chemotherapy, surgical resection, and/or radiation (45). The most common surgical option for local control is complete surgical resection of the tumor with reconstruction of the resulting defect, referred to as limb salvage surgery (LSS) (47, 48). While LSS has the main advantage of limb preservation, surgical decisions must be individually-based and are dependent on patient age, tumor location and size, expected growth plate involvement (and therefore anticipated limb length discrepancy), and desired functional outcome (3, 46, 49). To ensure adequate removal of the entire tumor, a cuff of surrounding normal tissue, often including adjacent muscle, tendon, fascia, and neurovascular structures, must be removed (3).

The most common site involved is the knee, affecting either the distal femur or proximal tibia. Wide resection often requires sacrifice of joint surfaces and/or adjacent ligamentous restraints, with a joint-replacing endoprosthesis that restores skeletal stability used as the most common form of reconstruction (3, 46). Due to its anatomic location, proximal tibial reconstruction requires augmentation of the soft tissue coverage and reconstruction of the patellar tendon extensor mechanism. This is most commonly done with a medial gastrocnemius rotational muscle flap positioned over the anterior portion of the tibial implant (3, 7, 49). In skeletally immature children, extendible endoprosthesis devices may be used to accommodate expected limb length discrepancies through sequential surgical or non-invasive lengthening procedures (47). For a more detailed information on surgical options and procedures, the reader is referred to other references for additional detail (3, 46–48, 50).

### Post-operative Medical Management and Rehabilitation

After surgery, individuals require careful monitoring for wound complications related to swelling and vascular compromise of tissue flaps and secondary infections related to the complexity of the surgical procedure and an immune-compromised state induced by chemotherapy and malnutrition in this population (48). Postoperative precautions include elevation, splinting and joint immobilization to facilitate wound healing, the duration of which can vary depending on the tumor location (femur vs. tibia), the type of endoprosthesis fixation used and other patient-specific factors. After distal femur reconstructions, post-operative knee immobilization is maintained until wound healing is accomplished, typically 2–3 weeks (51). Following proximal tibia reconstructions, a period of strict immobilization in knee extension is necessary to protect the reconstructed patellar tendon insertion during the healing phase, commonly around 6–8 weeks (13, 51). Early therapy interventions include active and passive ROM of the adjacent hip and ankle, along with transfer training and mobilization with partial or weight-bearing as tolerated while maintaining immobilization of the knee to protect the healing structures (13, 51). Rehabilitation is advanced after discontinuation of post-operative precautions with the goal of optimizing knee ROM, strength, balance, proprioception and functional gait.

## Impairments in Body Structure, Function, and Activity Are Common in Survivorship

### Common Impairments After Limb Salvage Procedure

Although some individuals achieve excellent function, many survivors experience residual impairments that impact function and activities of daily living. Common impairments include restrictions in ROM, strength deficits, and gait dysfunction (8, 9, 52). Impaired ROM, notably in knee flexion, is observed with two studies reporting an average flexion range between 106.1 and 109.3° in the surgical limb (compared to an average of 134.1 for the non-surgical limb) (6, 9). Importantly, while these studies included participants with both tibia and femur sarcomas, a separate study found patients with endoprosthesis management of proximal tibia tumors had a mean knee flexion ROM of only 60° (7). This difference may be due to the extended immobilization period required for healing of the knee extensor mechanism (13).

Strength deficits are also common, with ratios of surgical to non-surgical knee strength between 37.4 to 47.5% for extension and 54.5 to 71.7% for flexion (6). Strength deficits have also been observed in the non-surgical (contralateral) limb (53). A case series reports decreased unimpaired knee and hip muscle force production compared to normative values for knee flexion (74%), knee extension (63%), hip flexion (35%), and hip extension (13%) (12). Individuals with proximal tibia reconstructions have the additional challenge of achieving full active knee extension (the deficit of which is referred to as extensor lag) due to the necessary reconstruction of the distal insertion of the patellar tendon remnant onto the endoprosthesis after tumor resection (13).

Impairments in ROM and strength also contribute to gait dysfunction in this population. Spatiotemporal gait changes after LSS include decreased gait speed, stride length, and cadence, as well as changes to the time spent in single and double limb support (6, 12). The amount of soft tissue removed during surgery, knee extension strength, and knee flexion ROM are all predictive of impaired gait in individuals 1-year after LSS (10).

### Functional and Activity Impairments Limit Full Participation After LE Sarcoma Limb Salvage Procedures

Residual impairments after LSS may be expected to impact functional activities after the immediate and subacute rehabilitation period. Due to continued impairments, LSS patients may have difficulty with functional activities that are required for full participation in work, school or the community. Marchese et al. (9) examined functional mobility, QoL, and ROM in individuals after LSS and found significant correlations between ROM restrictions and functional and QoL measures (9). Additionally, performance limitations affect participation in childhood cancer survivors (CCS). In the Childhood Cancer Survivor Study, 29.1% of osteosarcoma survivors reported at least one limitation in physical ability, while 22.1% reported lingering pain from their medical condition (4). When compared to siblings, bone sarcoma CCS are 6.3 times more likely to have decreased attendance at school or work, with 11% reporting that poor health prevented their regular attendance (16). In addition, 51.8% of adult CCS managed with LSS are physically inactive and less likely to exercise compared to their siblings (54). When compared to other pediatric cancer diagnoses, survivors of bone sarcomas have the 2nd highest rate of performance limitations and participation restrictions, surpassed only by survivors of brain tumors (16). Although there have been vast improvements in medical and surgical management of bone sarcomas, the role and impact of muscle alteration remains largely unexamined in this population despite the likelihood that minimizing adverse effects of muscle dysfunction may have a significant role in improving function and participation in these individuals.

## Muscle Architecture Determines Muscle Function

### What Is Muscle Architecture and How Does It Relate to Muscle Function?

Muscle architecture refers to the size and orientation of muscle fibers and is highly predictive of muscle function (21). It is represented by a few key parameters, namely optimal fiber length (the length at which peak isometric tension is generated), physiological cross-sectional area (PCSA, a representation of the cross-sectional area (CSA) perpendicular to the muscle fibers), and the pennation angle (the angle of fiber insertion into the tendon) (22, 55–57). PCSA is distinct from purely anatomical cross-sectional measures in that the latter does not take into account the pennation or orientation of the fibers, and is usually in an anatomical plane rather than oriented in cross-section to the specific muscle's line of action. PCSA is the *strongest* predictor of optimal force generation in skeletal muscle, and therefore important to functional demands where optimal force is required (22, 57–60). While production of muscle force is complex, muscle architecture is one of the primary determinants of force production (21). Muscles important to human function vary with respect to their muscle architecture. For example, the soleus muscle, vital to posture and gait, has shorter fibers but one of the highest PCSAs in the lower limb (61). Conversely, the sartorius, a muscle with very long fibers related to its high lengthening capabilities, has a small PCSA and thus lower force-generating capabilities (61).

### How Is Muscle Architecture Normally Measured?

Direct measures of muscle architecture, such as muscle optimal fiber length and PCSA can be difficult to obtain, due to the need for several relatively invasive measures. Optimal fiber length requires not only direct knowledge of muscle fiber length but also concomitant measurement of sarcomere length. Fiber length is most commonly measured using direct dissection in either cadaveric specimens or utilizing muscle biopsy mechanisms, often during surgical procedures (21, 58, 62). Sarcomere length measurement has typically been performed on dissected fiber bundles or during surgery using laser diffraction methodologies (63–67), although newer techniques such as microendoscopy can measure sarcomere length *in vivo* without need for a surgical procedure (68, 69). However, microendoscopy still requires a needle insertion into the muscle to obtain measurements (70, 71). For these reasons, muscle fiber length is often *estimated* by using fascicle length as a proxy (21, 72).

Fascicle length, an important indicator of muscle excursion, is most commonly imaged with ultrasound (22, 73, 74). Ultrasound (US) is a validated technique that is accessible, cost-effective, and has been widely used for determining fascicle length in musculature across numerous populations (23, 38, 40, 72, 74–76). However, ultrasound imaging does not have the spatial resolution and field-of-view as other imaging modalities, including magnetic resonance imaging (MRI) and computed tomography (CT). In addition, operator-dependent error associated with excessive transducer pressure and/or probe orientation can occur, although careful utilization of reproducible methods enhances accuracy of this method (77–79). A 2013 systematic review concluded that ultrasound has good reliability for measurement of fascicle length across multiple conditions, and validity, although not as widely investigated, has also been reported as good, especially under reproducible conditions and during passive conditions (77).

Finally, PCSA is calculated using muscle volume measures *and* optimal fiber length. Whole muscle volume can be estimated using imaging (e.g., MRI), although may not take into account contractile content vs. other tissue (extracellular matrix, fat infiltration) within the muscle (59, 80, 81). In biological samples, PCSA is calculated using muscle mass (obtained by direct dissection) and muscle density measures from the literature and may take into account the pennation angle as well (21, 56, 58, 60, 82). Because of the complexity in obtaining the measures required for calculation of PCSA, measures of muscle size are often reported as anatomical CSA or volumetric measures. Multiple imaging modalities have been used to obtain surrogate measures of muscle size, including MRI, CT, and US (37, 83, 84). MRI provides excellent distinction between soft tissues and is well-suited for volume and CSA measurements. CT is also a valid and reliable modality for assessing muscle volume/CSA, but may not provide as much spatial resolution as MRI, and also comes with the risks of ionizing radiation. CT is faster and cheaper compared with MRI, although both have artifacts associated with metal implants that affect image quality (32, 85). Muscle thickness, as measured with US, can also provide an indicator of muscle size, although much like cross-sectional measures, this measurement will vary depending on the location of the image along the length of the muscle.

## Changes in Muscle Architecture Affect Human Function

### Changes in Muscle Size With Immobilization

Muscle CSA, specifically PCSA, is a strong indicator of force generation capacity, which is often limited in bone sarcoma childhood cancer survivors (CCS) (6, 8, 9, 12, 53, 86). We are unaware of any studies measuring any aspect of muscle size (PCSA, CSA, volume, or thickness) in this population. However, there are reports of changes in muscle size in populations related to the LSS population. Immobilization in particular is a potent catalyst for muscle atrophy, which occurs rapidly during periods of inactivity (33, 87–89). Seminal work in animal models demonstrated the dramatic effect of immobilization on a muscle's response, where animals immobilized with the soleus in a shortened position had significantly more atrophy than those immobilized in the lengthened position (90, 91). Interestingly, additional studies demonstrate that once immobilization is discontinued and normal joint function is returned, immobilization-induced changes can be reversed in as little as a few weeks (91–93). Although a return to normal muscle size and length would also be expected in healthy humans after immobilization, this may depend on the population and whether or not the normal catalyst for healthy muscle function can be restored. Research has shown that in healthy young males, a significant decrease (~3.5%) in quadriceps CSA occurred with as little as 5 days of leg immobilization, with even larger decreases (~8.4%) in those immobilized for 14 days (33). Concurrent decreases in strength of 9.0% and 22.9% with 5 and 14 days of immobilization also occurred (33). Given the immobilization and inactivity that occurs after limb salvage surgeries, it is expected that muscle atrophy could be one factor in the strength deficits seen in this population, especially given the concomitant medical therapies and altered function that this population experiences.

### Evidence for Muscle Atrophy After Surgery

Atrophy has been observed after knee surgery, as reported in total knee arthroplasty (TKA) and ACL reconstruction (ACLR). After TKA, studies have documented reductions in muscle volume between 5 to 20%, with ~10% reduction seen within the first month (30, 41). This atrophy explains much of the variance in strength deficits after TKA, with impaired voluntary activation another important factor (41, 94). Similarly, reductions in quadriceps voluntary activation and muscle CSA/volume occur within 12 weeks after ACLR, with atrophy accounting for almost half of observed strength deficits; neuromuscular recruitment deficits also likely contribute during this timeframe (26). Atrophy has been shown to impact function, with quadriceps weakness linked to fall risk, decreased gait speed, and stair-climbing difficulty (30).

While voluntary activation deficits greatly impact muscle strength in the acute timeframe, there is evidence that atrophy plays a larger role later in recovery. Studies report quadriceps central activation ratio near normal (>90%) at both 3 and 6-months post-surgery, and that decreased quadriceps CSA explained more of the variance in strength than did activation deficits after ACLR (29, 43). In individuals cleared to return to activity post-ACLR, researchers found that partial quadriceps volume was significantly and strongly correlated (*r* = 0.830, *p* < 0.001) with knee extension strength (95). Furthermore, there is evidence that differences in quadriceps muscle thickness exist even 2-years post-ACLR (96). Although much of this evidence is limited to the adult population, post-operative muscle atrophy and activation deficits are also likely to occur in the LSS population.

### Changes in Fascicle Length Affect Muscle Properties and Function

Changes in fascicle length have been observed in various populations and have a substantial effect on total muscle excursion as well as contraction velocity. Shorter fascicles have been observed in the gastrocnemius, biceps brachii, and brachialis in adults post-stroke, potentially due to the limited active ROM available in this population (37–39). In addition, changes in fascicle length in the gastrocnemius after stroke have been shown to alter the active force-length properties of that muscle (39). Shorter fascicles have also been observed in the biceps femoris in adults with history of ACLR (97). Although altered fascicle length cannot *independently* explain all changes in force-generating characteristics, concurrent changes in sarcomere length and number, both of which have been reported in animal and human studies, could have implications on generation of muscle force (91–93, 98, 99). Importantly, muscle excursion and the length at which it is immobilized has been shown to be an important stimulus for muscle growth by serial sarcomere addition (92, 100, 101). Although there is limited evidence of fascicle or sarcomere length changes in populations similar to LSS, limitations in active joint function could also be expected to result in fascicle length and/or sarcomere changes in the LSS population and could potentially affect force-length properties.

### Potential Muscle Architectural Changes in Individuals With Bone Sarcoma

Due to the biologic and intrinsic changes in muscle as a result of chemotherapy and radiation, muscle architectural changes may amplify continued dysfunction after an individual undergoes surgery. Chemotherapy and radiation have disruptive and destructive effects on not only cancerous but also healthy cells, including skeletal muscle (102, 103). Through oxidative stress, DNA damage, loss of mitochondria and satellite cell function, and alterations in blood vessel perfusion, chemotherapy and radiation induce muscle atrophy and impaired muscle performance (102–108). Studies in adult survivors of breast cancer and animal models demonstrate that exercise can mitigate these negative effects, although it is unclear if this occurs in the same manner in a skeletally immature population such as in LSS (109–112).

**Figure 1:**
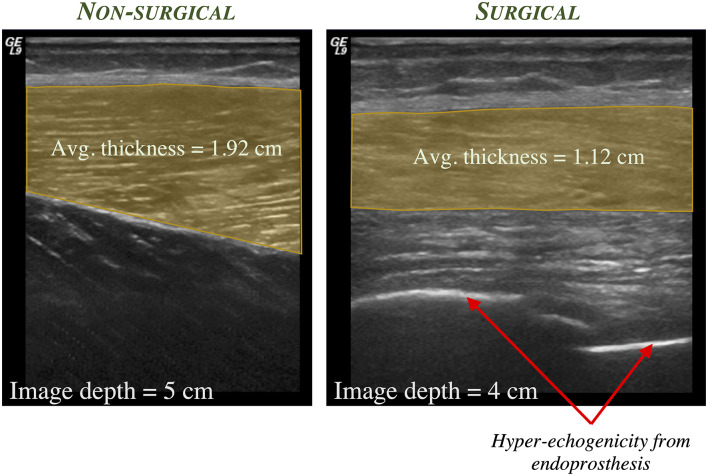
Representative static 2-D ultrasound images of the anterolateral thigh in a 17-years old male who was diagnosed at age 11 with osteosarcoma of the distal femur and underwent subsequent limb salvage surgery with Stanmore endoprosthesis placement and lengthening. The thickness of the vastus lateralis muscle (shaded region) is greater in the non-surgical limb (left) compared to the surgical limb (right).

**Figure 2:**
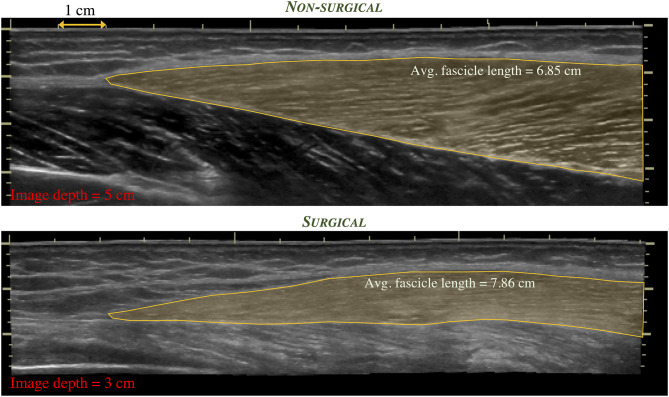
Extended field-of-view ultrasound images depicting the distal region of the vastus lateralis muscle in the same individual as in Figure 1. Differences in regional muscle thickness, fascicle orientation, and length (shaded region) can be appreciated between the non-surgical limb (top) and the surgical limb (bottom).

Finally, limb length discrepancies, common in skeletally-immature children, may increase functional deficits in this population. Muscle is capable of rapidly adapting to changes in limb length, as with limb lengthening. A case report described changes in fascicle length, sarcomere length, and sarcomere number in a 17-years old female who underwent a limb lengthening procedure due to an arrest of her femoral growth plate (98). Rapid increase in fascicle length during the distraction period along with a decrease in sarcomere length were reported, demonstrating an increase in sarcomere number that exceeds that expected by the femur length change. In the LSS population, it is anticipated that both muscle thickness and fascicle length may change given the surgical procedure and post-operative rehabilitation protocols. Preliminary evidence demonstrates this possibility, as shown in Figures 1, 2 in a 17-years old after endoprosthesis reconstruction for a distal femur osteosarcoma.

## Discussion and Future Directions for Research

Effective management of bone sarcomas has led to improved survival and an increasing population of LSS survivors. However, individuals continue to experience long-term impairments and functional deficits even years after treatment. Impaired muscle function may be due to multiple factors, including chemotherapy and/or radiation, difficulties with muscle activation, and surgical interruption of various soft tissues, especially after muscle flaps and extensor mechanism reconstructions. Muscle architecture's significant role in muscle function warrants continued investigation into these changes and their effect on muscle properties in this population.

Studies are needed to investigate the mechanisms and anatomic determinants of muscles affected after LSS. Ultrasound may provide crucial information about muscle architecture as it relates to function in this unique population. Changes in muscle architecture as seen with immobility, may drive secondary adaptations in muscles primarily responsible for activities leading to alterations in gait and overall function. Lastly, how these architectural changes affect force production and function would provide insight that may guide surgeons as they perform these complex, life-saving procedures.

Rehabilitation after LSS, which is not currently standardized, focuses primarily on functional improvement, protection of healing tissues, education on potential modifications to commonly performed functions, strengthening and ROM. Although weightbearing and ROM restrictions and precautions may be unavoidable, specific knowledge on how muscle function is impacted would be valuable in designing specific rehabilitation programs. For instance, as alterations in fascicle length have been shown to change the shape of the force-length curve in the stroke population, changes in muscle architecture in the sarcoma population as well as continued growth in this younger population would be anticipated to alter these important muscle properties. Clinical knowledge of these processes could help tailor exercise and strengthening programs to minimize, reverse or improve properties in specific musculature that are most affected and important to functional activities after surgical procedures.

Finally, the long-term significance of these potential architectural changes for these individuals cannot be understated. Knowledge of potential muscle architectural ramifications to function should be considered in order to optimize patient-specific outcomes. The use of novel imaging techniques, biomechanical models, and collaborative clinical care may restore function so that these individuals can return to activities that are important to their well-being and quality of life.

## Data Availability Statement

The datasets generated for this study are available on request to the corresponding author.

## Ethics Statement

The studies involving human participants were reviewed and approved by Institutional Review Board of University of Maryland, Baltimore. Written informed consent to participate in this study was provided by the participants' legal guardian/next of kin.

## Author Contributions

CN, VM, and OA contributed to the conception and design of this review. KR and CN performed the primary literature search. All authors contributed to development of the final submitted manuscript.

## Conflict of Interest

The authors declare that the research was conducted in the absence of any commercial or financial relationships that could be construed as a potential conflict of interest.

## Abbreviations

ACLR: ACL reconstruction
CCS: childhood cancer survivor
CT: computed tomography
LE: lower extremity
LSS: limb salvage surgery
MRI: magnetic resonance imaging
ROM: range of motion
TKA: total knee arthroplasty
US: ultrasound.
